# The General Transcription Repressor *TaDr1* Is Co-expressed With *TaVrn1* and *TaFT1* in Bread Wheat Under Drought

**DOI:** 10.3389/fgene.2019.00063

**Published:** 2019-02-08

**Authors:** Lyudmila Zotova, Akhylbek Kurishbayev, Satyvaldy Jatayev, Nikolay P. Goncharov, Nazgul Shamambayeva, Azamat Kashapov, Arystan Nuralov, Ainur Otemissova, Sergey Sereda, Vladimir Shvidchenko, Sergiy Lopato, Carly Schramm, Colin Jenkins, Kathleen Soole, Peter Langridge, Yuri Shavrukov

**Affiliations:** ^1^Faculty of Agronomy, S.Seifullin Kazakh AgroTechnical University, Astana, Kazakhstan; ^2^Institute of Cytology and Genetics, Siberian Branch of the Russian Academy of Sciences, Novosibirsk, Russia; ^3^A.F.Khristenko Karaganda Agricultural Experimental Station, Karaganda, Kazakhstan; ^4^Biological Sciences, College of Science and Engineering, Flinders University, Bedford Park, SA, Australia; ^5^School of Agriculture, Food and Wine, University of Adelaide, Adelaide, SA, Australia; ^6^Wheat Initiative, Julius Kühn-Institut, Berlin, Germany

**Keywords:** Amplifluor-like SNP marker, bioinformatics, drought, general repressor of transcription, *TaDr1*, *TaFT1*, *TaVrn1*

## Abstract

The general transcription repressor, *TaDr1* gene, was identified during screening of a wheat SNP database using the Amplifluor-like SNP marker KATU-W62. Together with two genes described earlier, *TaDr1A* and *TaDr1B*, they represent a set of three homeologous genes in the wheat genome. Under drought, the total expression profiles of all three genes varied between different bread wheat cultivars. Plants of four high-yielding cultivars exposed to drought showed a 2.0–2.4-fold increase in *TaDr1* expression compared to controls. Less strong, but significant 1.3–1.8-fold up-regulation of the *TaDr1* transcript levels was observed in four low-yielding cultivars. *TaVrn1* and *TaFT1*, which controls the transition to flowering, revealed similar profiles of expression as *TaDr1*. Expression levels of all three genes were in good correlation with grain yields of evaluated cultivars growing in the field under water-limited conditions. The results could indicate the involvement of all three genes in the same regulatory pathway, where the general transcription repressor *TaDr1* may control expression of *TaVrn1* and *TaFT1* and, consequently, flowering time. The strength of these genes expression can lead to phenological changes that affect plant productivity and hence explain differences in the adaptation of the examined wheat cultivars to the dry environment of Northern and Central Kazakhstan. The Amplifluor-like SNP marker KATU-W62 used in this work can be applied to the identification of wheat cultivars differing in alleles at the *TaDr1* locus and in screening hybrids.

## Introduction

Amongst the many types of abiotic stresses, drought or water limitation is one of the most important challenges for native plants and crops. There are several genetic and breeding strategies aimed at improving tolerance to drought in crops (Reviewed in: Ingram and Bartels, 1996; Yordanov et al., 2000; Tuberosa and Salvi, 2006; Valliyodan and Nguyen, 2006; Shanker et al., 2014; Berger et al., 2016; Kaur and Asthir, 2017). One potential approach is the modulation of flowering time, where wheat plants grow faster and complete their life-cycles a few days earlier, therefore minimizing interruption from oncoming, terminal drought (Reviewed in: Shavrukov et al., 2017). Genetic polymorphism and the introgression of novel alleles from wheat progenitors, relatives and wild species from the genus *Triticum* is a very powerful tool to enrich the genome of modern cultivars (Reviewed in: Arzani and Ashraf, 2017; Mwadzingeni et al., 2017; Wang et al., 2018).

Molecular markers are used widely for the identification of novel and existing alleles, and to track specific alelles in elite wheat breeding lines and introgression from landraces or wild species. Analysis of SNP (Single nucleotide polymorphism) is a rapidly developing technology with a diverse range of methods and applications (Reviewed in: Schramm et al., 2019). Amplifluor SNP markers are well-established and have been successfully applied in the recent genotyping of candidate genes for various plant species (Absattar et al., 2018; Yerzhebayeva et al., 2018; Khassanova et al., 2019). This includes research in bread wheat, where alleles of candidate genes for drought tolerance, *TaDREB5* and *TaNFYC-A7*, were identified using Amplifluor SNP markers. These genes demonstrate differential expression in high- and low-yielding wheat cultivars from Kazakhstan under a progressive drought and rapid dehydration (Shavrukov et al., 2016b; Zotova et al., 2018). In other studies, over-expression of transcription factors, *TaNFYA-B1* and *TaNF-YB3;l* showed increased yield and nitrogen uptake, and quicker root development and improved tolerance to drought than controls, respectively (Qu et al., 2015; Yang et al., 2017). Similarly, the rice genes *OsNF-YA7* and *OsNF-YB1* were reported to be responsive to drought. Over-expression of *OsNF-YA7* increased drought tolerance in transgenic rice plants (Lee et al., 2015), and *OsNF-YB1* controls grain filling, resulting in improved yield (Xu et al., 2016).

Transcription factor (TF) Nuclear Factor Y (NF-Y) is a synonym of CCAAT Binding Factor (CBF) and Heme Activator Protein (HAP). Three subunits (A, B, and C) usually function in a single protein complex of NF-Y, and each of the three components is essential for binding to *cis*-elements in the promoter regions of target genes (Siefers et al., 2009; Petroni et al., 2012). In plants, the functions of NF-Y proteins are quite diverse, but, for the purposes of this paper, we will focus on just three: (1) regulation of flowering time; (2) response to abiotic stress, particularly drought; and (3) overall productivity in different plants (Gusmaroli et al., 2001; Nelson et al., 2007; Petroni et al., 2012; Kuromori et al., 2014; Swain et al., 2017; Zhao et al., 2017) including bread wheat (Qu et al., 2015; Yadav et al., 2015; Zotova et al., 2018).

In *Arabidopsis*, the C subunits of NF-Y factor, AtNF-YC3, AtNF-YC4, and AtNF-YC9, are involved in the regulation of photoperiod-mediated flowering time through the GA signaling pathway by binding to RGA (Repressor of ga1-3) and RGL2 (RGA-like2) proteins (Hou et al., 2014; Liu et al., 2016). Over-expression of many individual NF-YC subunits (such as NF-YC1, NF-YC2, NF-YC3, NF-YC4, and NF-YC9) alters flowering time. Individual subunits of the NF-Y complex can affect the transcript levels of *Flowering locus T* (*FT*). This gene encodes the protein that is the key integrator in the flowering time pathway, and up- or down-regulation of FT in interaction with the NF-Y complex, leads to either early or late flowering in *Arabidopsis* (Kumimoto et al., 2010; Cao et al., 2014; Hou et al., 2014; Xu et al., 2016).

The flowering time trait has a complicated, multi-level control. Transcriptional up-regulation of two genes, *Vrn* (Vernalisation) and *FT*, is strongly required for the transition from the vegetative to reproductive stage, largely determining time to flowering (Reviewed in: Greenup et al., 2009; Jung and Müller, 2009; Yan, 2009; Jarillo and Piòeiro, 2011; Song et al., 2013; Milec et al., 2014; Blümel et al., 2015). In wheat, one of the most important crops, the genetic control of the flowering time trait has been extensively studied (Reviewed in: Li and Dubcovsky, 2008; Craufurd and Wheeler, 2009; Distelfeld et al., 2009; Campoli and Korff, 2014; Kamran et al., 2014). The main regulatory control of flowering time in wheat is through the up-regulation of *TaFT1* – *TaVrn3* and *TaVrn1* genes (Li and Dubcovsky, 2008; Distelfeld et al., 2009).

Interestingly, flowering time is controlled not only by genes during ontogenesis, but is strongly impacted by abiotic stresses (Reviewed in: Kazan and Lyons, 2016; Takeno, 2016). Plants of various species have been reported to alter their development and flowering time in response to different types of abiotic stresses, ranging from osmotic stress in *Arabidopsis* (Chen et al., 2007), to soil pH in a native population of *Corydalis sheareri*, Papaveraceae (Huang et al., 2017). However, drought has been shown to be one of the major abiotic factors affecting development of flowering in various plant species such as tea, *Camellia sinensis* (Sharma and Kumar, 2005), litchi, *Litchi chinensis* (Shen et al., 2016) and lemon (Li et al., 2017). The genetic control of reproductive development and time to flowering in response to various abiotic stresses are well studied in cereals (Gol et al., 2017), where the influence of cold (Li et al., 2018) and drought (Pinto et al., 2010; Gudys et al., 2018) in particular, affect grain yields. Early flowering as a drought escape strategy in wheat and other species and was reviewed recently (Shavrukov et al., 2017).

In bread wheat, the *TaVrn1* gene was mapped to the long arm of chromosome 5A, tightly linked with the *Q* gene controlling spike morphology (Kato et al., 1998). The *Q* gene belongs to the large *AP2/ERF* family of TF (Konopatskaia et al., 2016), which includes *DREB* genes responsive to drought and dehydration, and reports have shown that the *Q* gene is also regulated by drought (Gürsoy et al., 2012). Therefore, flowering time and spike morphology seem to have a shared regulatory framework with *TaVrn1* and *Q* genes, and a strong response to drought.

The gene sequence and structure of the general repressor of transcription, *Dr1* (alternative name – *NC2β*), is conserved among various eukaryotes. It operates as a heterodimeric complex with the product of another gene, *DrAP1* (alternative name – *NC2α*), and strongly represses the transcriptional activity of RNA polymerase II and III, but not RNA polymerase I (Kim et al., 1997). Originally, Dr1/DrAp1 was identified in human cells as an unknown factor that was able to inhibit TBP-dependent basal transcription *in vitro* (Inostroza et al., 1992). Mammalian DrAp1 itself cannot repress transcription and therefore it is considered as an enhancer of Dr1 repression activity (Mermelstein et al., 1996; Kim et al., 1997; Yeung et al., 1997). In *Drosophila*, Dr1/DrAp1 represses the transcription from TATA-containing promoters and activates the transcription from promoters without TATA-boxes (Willy et al., 2000).

In plants, *Dr1* was originally discovered in *Arabidopsis* (Kuromori and Yamamoto, 1994). Later, the rice *OsDr1* and *OsDrAp1* genes were cloned, and formation of the heterodimeric complex, interaction of the protein complex with DNA, and repressive activities of the subunits and protein complex were characterized using the Y2H system, *in vitro* methods, and a transient expression assay (Song et al., 2002). These authors demonstrated several differences between the properties of Dr1 and DrAp1 in mammals and rice. Firstly, the plant DrAp1 protein was found to be larger than the mammalian and yeast proteins, and both plant Dr1 and DrAp1 contained a greater number of domains/motifs than their mammalian counterparts. Secondly, OsDrAp1 alone showed stronger repression activity than OsDr1, therefore in plants, OsDr1 most likely plays the co-repressor role and enhances the activity of OsDrAp1 (Song et al., 2002).This differs from mammals and yeast, where Dr1 is the repressor and DrAp1 plays the role of a regulatory subunit (Inostroza et al., 1992; Kim et al., 1997; Prelich, 1997).

Two homologs *Dr1* genes from bread wheat, *TaDr1A* and *TaDr1B*, were identified and their expression patterns were reported in different wheat tissues under control and drought conditions (Stephenson et al., 2007). Transcripts of both *TaDr1* homologs were abundant in all tested plant tissues and strongly up-regulated in leaves under drought.

In yeast, a 71% similarity between Dr1 and CBF-A (=NF-YB) was reported (Sinha et al., 1996). In bread wheat, TaDr1 and TaDr2 proteins (accessions AF464903 and BT009234, respectively), showed a “high degree of similarity” with TaNF-YB3 amino acid residues (Stephenson et al., 2007). Therefore, the authors suggested that the Dr1/DrAp1 complex could, potentially, inhibit transcription by acting as antagonist to all or to particular NF-YB and NF-YC subunits, thus preventing subunit association and subsequent binding of the activation NF-Y complex (Stephenson et al., 2007). This could be a possible mechanism to explain *TaDr*-mediated global repression of transcription.

The aims of this work were: (1) to compare flowering time and time to grain maturity of high-yielding and low-yielding wheat cultivars from Kazakhstan; (2) to analyze the genetic polymorphism of the *TaDr1* gene in eight selected bread wheat cultivars, and in an F_3_ segregating population 18-6 originating from a complex interspecies hybridisation; (3) to study *TaDr1*, *TaVrn1* and *TaFT1* gene expression in response to drought in leaves of selected wheat cultivars; and (4) to assess the co-expression of *TaDr1*, *TaVrn1*, and *TaFT1* genes and grain yields of wheat cultivars in the dry conditions of Northern and Central Kazakhstan.

## Materials and Methods

### Plant Material, Conditions of Plant Growth and Drought Application

Eight wheat cultivars, representing two groups with contrasting yields were selected from local varieties tested in field trials, based on their grain yields under the dry conditions in Northern Kazakhstan (current study) and Central Kazakhstan, described earlier by Shavrukov et al. (2016b). Descriptions of plant materials and all experiments were as reported earlier (Zotova et al., 2018). These descriptions included: seeds obtained, conditions of plant growth in the research field in Central Kazakhstan and the controlled conditions in the “Phytotron” experiments on gradual drought using plants in soil-filled containers over 12 days (Experiment 1) (Zotova et al., 2018).

A small outdoor trial was conducted in the research field of S.Seifullin Kazakh AgroTechnical University, Astana in Northern Kazakhstan in the dry season of 2017. Total rainfall was 107 mm during the vegetative growth period, lower than the average of 166 mm that was observed over many years in this region, and a 3°C higher than average temperature for August (20.3°C compared to the average, 17.3°C) was recorded that year. Two-row plots were sown, 1 m in length with 5 cm between plants in rows and 20 cm between rows, and four randomized replicates were used. The number of days between sowing and first flowering of 50% of plants in each plot was counted as “Days to flowering” (DF), while “Days to maturity” (DM) was recorded when all plants in each plot reached the ripening stage. Grain yield was measured for each plot and re-calculated in “g/m^2^” with statistical treatment as described below.

A complex interspecific cross [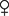 *Triticum spelta*, k-53660 × 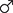 (*T. aestivum*, Novosibirskaya 67 / T. *dicoccum*, k-25516)] was produced by one of the authors, Nikolay Goncharov, at the Institute of Cytology and Genetics, Russian Academy of Sciences, Novosibirsk (Russia). F_3_ plants from the hybridisation were grown in pots with soil in a “Phytotron” with controlled conditions as mentioned above.

### Identification of the “Gene of Interest” Using Bioinformatics and Molecular Phylogenetic Comparative Analysis

The cereals SNP database^1^ was used to search and select a single target gene or “Gene of Interest” (GoI) for further research. BLAST analysis of the genetic fragments containing a SNP was applied to identify the full-length GoI using the Nucleotide collection of bread wheat in the NCBI database^2^.

Bioinformatics and systems biology methods were applied in this study to identify the full-length nucleotide sequence of the GoI, *TaDr1*, and its corresponding polypeptide sequence was used for both BLASTN and BLASTP in NCBI and in GenomeNet Database Resources, Kyoto University, Japan^3^. All wheat gene sequences with KEGG identification and their encoded proteins were retrieved from GenomeNet databases. Multiple sequence alignments of nucleotide sequences for the *TaDr1A* and *TaDr1B* genes were conducted in CLUSTALW using the CLC Main Workbench software^4^. Chromosome locations of all *TaDr1* homeologous genes in the wheat genome were found using BLAST analysis with high confidence annotated genes of the IWGSC database at the Gramene web-site^5^.

The molecular dendrogram of polypeptides of *TaDr1* from bread wheat and other monocot plants was constructed using SplitsTree4 program^6^ (Huson and Bryant, 2006), with Phylogram Splits and Tree Selector option.

### DNA Extraction and SNP Amplifluor Analysis

Plants were grown in control (non-stressed) conditions in containers with soil as described above. Five uniform, 1 month-old individual plants were selected from each accession and five leaves were collected and bulked for leaf samples. Leaf samples frozen in liquid nitrogen were ground in 10-ml tubes with two 9-mm stainless ball bearings using a Vortex mixer. DNA was extracted from the bulked leaves with phenol-chloroform as described in our earlier papers (Shavrukov et al., 2016b; Zotova et al., 2018). 1 μl of DNA was loaded on a 0.8% agarose gel to assess quality, and concentration was measured by Nano-Drop (ThermoFisher, United States).

Amplifluor-like SNP analysis was carried out using a QuantStudio-7 Real-Time PCR instrument (ThermoFisher Scientific, United States) as described previously (Jatayev et al., 2017; Zotova et al., 2018) with the following adjustment for wheat genotyping. Each reaction contained 3 μl of template DNA adjusted to 20 ng/μl, 5 μl of Hot-Start 2xBioMaster (MH020-400, Biolabmix, Novosibirsk, Russia^7^) with all other components as recommended by the manufacturers, including MgCl_2_ (2.0 mM). One μl of the two fluorescently labeled Universal probes was added (0.125 μM each) and 1 μl of allele-specific primer mix (0.075 μM of each of two forward primers and 0.39 μM of the common reverse primer). 4 μl of Low ROX (ThermoFisher, United States) was added as a passive reference label to the Master-mix as prescribed for the qPCR instrument. Assays were performed in 96-well microplates. The annotated SNP sites were used to design allele-specific primers. Sequences of the Universal probes and primers and sizes of amplicons generated are presented in Supplementary Material 1.

PCR was conducted using a program adjusted from those published earlier (Jatayev et al., 2017; Zotova et al., 2018): initial denaturation, 95°C, 2 min; 20 “doubled” cycles of 95°C for 10 s, 60°C for 10 s, 72°C for 20 s, 95°C for 10 s, 55°C for 20 s and 72°C for 50 s; with recording of Allele-specific fluorescence after each cycle. Genotyping by SNP calling was determined automatically by the instrument software, but each SNP result was also checked manually using amplification curves and final allele discrimination. Experiments were repeated twice over different days, where two technical replicates confirmed the confidence of SNP calls.

### RNA Extraction, cDNA Synthesis and qPCR Analysis

Plants were grown in the controlled conditions of a “Phytotron” at S.Seifullin Kazakh AgroTechnical University, Astana, Kazakhstan, as described earlier in Experiment 1 (Zotova et al., 2018). In brief, for mild drought stress with 1-month old plants, watering was withdrawn in one of two soil-filled containers for 12 days until wilted leaves were observed. Control plants in similar containers were watered continuously. Five individual plants were used for each cultivar in drought-affected and well-watered containers. All leaves were collected from each plant in plastic tubes as separate biological replicates, frozen immediately in liquid nitrogen and kept at -80°C until RNA extraction. Three samples were used for RNA extraction in each cultivar and treatment, while two additional samples were used as replacements in case of failed extraction or poor RNA quality.

Frozen leaf samples were ground as described above for DNA extraction. TRIzol-like reagent was used for RNA extraction following the protocol described by Shavrukov et al. (2013) and all other steps for RNA extraction and cDNA synthesis were as described previously (Zotova et al., 2018) including DNase treatment (Qiagen, Germany), and the use of a MoMLV Reverse Transcriptase kit (Biolabmix, Novosibirsk, Russia). The quality of all cDNA samples was confirmed by PCR with products of the expected size.

Samples of cDNA diluted with water (1:2) were used for qPCR analyses using both a QuantStudio-7 Real-Time PCR instrument (ThermoFisher Scientific, United States) at Kazakh AgroTechnical University, Astana, Kazakhstan, and Real-Time qPCR system, Model CFX96 (BioRad, Gladesville, NSW, Australia) at Flinders University, Australia. Similar qPCR protocols were used in both instruments, as described earlier (Shavrukov et al., 2016b). Differences between protocols were: the total volume of 10 μl q-PCR reactions included either 5 μl of 2xBiomaster HS-qPCR SYBR Blue (Biolabmix, Novosibirsk, Russia) for experiments in Kazakhstan or 5 μl of 2xKAPA SYBR FAST (KAPA Biosystems, United States) for experiments in Australia, 4 μl of diluted cDNA, and 1 μl of two gene-specific primers (3 μM of each primer) (Supplementary Material 2). Expression data for the target genes were calculated relative to the average expression of the two reference genes: *Ta22845*, ATP-dependent 26S proteasome and *Ta54825*, actin (Paolacci et al., 2009). At least three biological and two technical replicates were used in each qPCR experiment.

### Statistical Analysis

IBM SPSS Statistical software was used to calculate and analyze means and standard error using ANOVA, to estimate the probabilities for significance using Student’s *t*-test. A correlation analysis was performed using Tests of Between-Subjects Effects (IBM SPSS, Statistics Desktop 25.0.0.0).

## Results

### Phenological Characteristics and Grain Yield of Studied Wheat Cultivars

To assess the relative grain yield performance of the bread wheat cultivars in the dry conditions of Northern and Central Kazakhstan, eight wheat cultivars were selected from our previously published paper (Shavrukov et al., 2016b), and tested in the field during the dry season of 2017. The group of four cultivars (1. Aktyubinka; 2. Albidum 188; 3. Altayskaya 110; and 4. Saratovskaya 60) performed as expected, confirming their high-yielding status, which was significantly higher than the group with low-yield (5. Vera; 6. Volgouralskaya; 7. Yugo-Vostochnaya 2; and 8. Zhenis) (Table 1).

**Table 1:** Phenological characteristics of eight wheat cultivars grown in the Akmola region, Northern Kazakhstan, in the dry season of 2017.

		Days to	Days to	Grain yield
Group	Cultivar	flowering	maturity	(g/m^2^)
High-yield	Aktyubinka	39	66	240 ± 14^a^
	Albidum 188	42	66	165 ± 11^b^
	Altayskaya 110	42	68	155 ± 10^b^
	Soratovskaya 60	40	66	162 ± 10^b^
**Average of the high-yielding group**		40.8 ± 0.9^∗^	66.5 ± 0.6	180.5 ± 23.0^∗^
Low-yield	Vera	43	67	129 ± 9^c^
	Volgouralskaya	43	74	122 ± 9^c^
	Yugo–Vostoch. 2	42	65	112 ± 8^c^
	Zhenis	45	67	129 ± 7^c^
**Average of the low-yielding group**		43.3 ± 0.7^∗^	68.3 ± 2.3	123.0 ± 4.3^∗^

*Number of Days to flowering (DF) was counted when 50% of plants in the plot started flowering, while number of Days to maturity (DM) was recorded once all plants in each plot reached the ripening stage. Grain yield was calculated in g/m^2^, as average of four replicates ± SE. Different letters in superscripts and asterisks (^∗^) indicate significant differences (*p* < 0.05) using ANOVA*.

The superior high-yielding cultivar Aktyubinka (240 g/m^2^) had the shortest DF (39 days) and so earliest start to flowering, while its DM was about average for this group (66 days). In contrast, the lowest-yield cultivar, Yugo-Vostochnaya 2, with more than two-fold lower grain yield than Aktyubinka, started flowering after a 3 day delay (42 days) but was only 1 day shorter in DM (65 days) compared to Aktyubinka. On average, the four high-yielding cultivars started flowering a significant 2.5 days earlier compared to the low-yielding group, while a less pronounced and insignificant difference (1.8 days) was found in DM between the two groups of cultivars (Table 1).

### Genotyping of Wheat Accessions for the *TaDr1* Gene Using an Amplifluor SNP Marker

**Figure 1:**
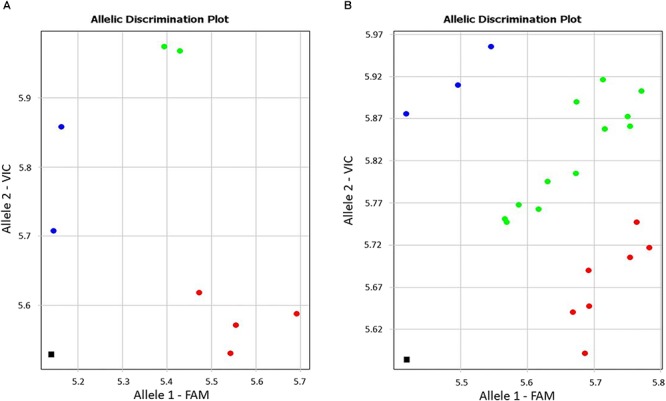
Allele discrimination in eight wheat cultivars **(A)** and in the segregating population 18-6 **(B)** using the Amplifluor-like SNP marker KATU-W62. *X*- and *Y*-axes show relative amplification units, ΔR_n_, for FAM and VIC fluorescence signals, respectively. Red dots represent homozygote (*aa*) genotypes with allele 1 (FAM) associated with the high yielding cultivars, blue dots represent homozygote (*bb*) genotypes for allele 2 (VIC), and green dots represent heterozygote (*ab*) or mixed genotypes identified with automatic SNP calling. The black squares show the no template control (NTC) using water instead of template DNA.

During screening of annotated SNPs in bread wheat, the contig BC000036325 was identified for the drought-responsive candidate gene (*TaDr1*) using the publicly available database Cereal DB (see text footenote 1). The SNP marker KATU-W62 was developed to target the annotated SNP [W = A/T] in the 3′-UTR (untranslated region) based on the sequence of BC000036325. Both selected wheat cultivars and the segregating population 18-6 showed genetic polymorphism, with the more common allele being the nucleotide “A” and rarer allele “T” at the SNP position (Figure 1).

Genotyping of plants from the eight studied cultivars using the Amplifluor SNP marker KATU-W62 revealed clear discrimination of homozygote genotypes “*aa*” in all four high-yielding cultivars (1–4) while low-yielding cultivars (5–8) were characterized by a mixture of “*bb*” (**5.** Vera; and **7.** Yugo-Vostochnaya 2) and “*ab*” (**6.** Volgouralskaya; and **8.** Zhenis) genotypes (Figure 1A). At this stage, it remains unclear whether the “*ab*” genotypes of cultivars Volgouralskaya and Zhenis belong to true heterozygotes, a mixture of several genotypes or both cases together.

Segregation of genotypes for the SNP marker KATU-W62 was observed in the F_3_ population 18-6 (Figure 1B) originating from the complex cross, where the favorable allele “*a*” was inherited from the paternal line. The analysis of the entire hybrid population is still ongoing and will include progeny analyses in the next generation.

### Bioinformatic Characterisation of the *TaDr1* Candidate Gene and Protein

BLASTN results at NCBI^8^ for bread wheat gene sequences revealed two accessions, BT009234 for *TaDr1B*, and AF464903 for *TaDr1A*, published and described earlier (Stephenson et al., 2007), with 96% identity in both genes, and covering 96% and 89% of the sequences, respectfully.

Genomic DNA analysis using high confidence genes annotated by the IWGSC database revealed that *TaDr1A* and *TaDr1B* are located on homeologous chromosomes 3A and 3D, in the positions 689,352,814-689,357,320 and 552,949, 442-552,953,939, on the forward strands of the physical map, respectively. These genes, TraesCS3A02G450700 and TraesCS3D02G443500, contained five exons, produced 1,536 and 1,565 bp long transcripts which encoded 291 and 298 amino acid long proteins, respectively. The sequence of contig BC000036325, which contained the identified SNP, had the highest level of identity (99.7%) with the gene TraesCS3B02G487800, located in the position 733,818,973-733,823,767, on the forward strand of the physical map of the homeologous chromosome 3B. The gene presented in the BC000036325 contig also contained five exons, transcribed a single 1,317 bp long transcript and encoded a 296 amino acid long protein. Therefore, the two annotated genes *TaDr1A* and *TaDr1B*, and the BC000036325 contig from the SNP database, together represent the three homeologous genes of *TaDr1* in wheat genomes A, D and B, respectively.

**Figure 2:**
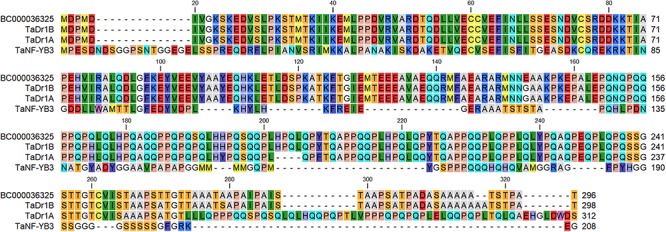
BLASTP protein comparison of the annotated sequence BC000036325 (http://www.cerealsdb.uk.net) with two forms of the general repressor of transcription, TaDr1B (BT009234) and TaDr1A (AF464903), and the TF TaNF-YB3 (BT009265), presented using CLC Main Workbench software.

The protein encoded by BC000036325 shared 99.3% and 85.% identity with TaDr1B and TaDr1A, respectively, while a low similarity score and only 18.9% identity was found compared to TaNF-YB3, accession BT009265 (Figure 2). This result shows that accession BC000036325 from the B genome used in this work has much stronger similarity to TaDr1B and to the corresponding gene *TaDr1B* from the D genome of wheat.

### Molecular Dendrogram of the *TaDr1* Gene

**Figure 3:**
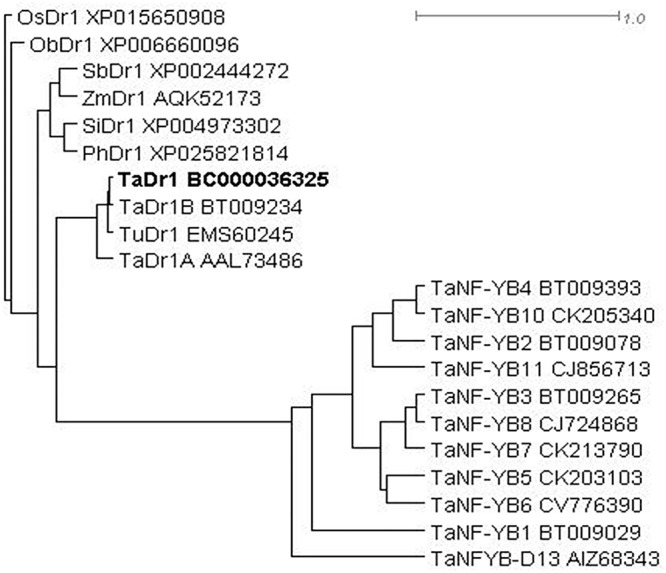
Molecular phylogenetic tree of proteins encoded by *Dr1* genes in monocot plants with the comparison to peptide sequences of TaNF-YB TFs in wheat. Rooted BioNJ dendrogram was generated by program SplitsTree4 (Huson and Bryant, 2006; http://www.splitstree.org). Scale bar shows uncorrected P genetic distance equivalent to 1.0. Accession sequences were retrieved from NCBI database. Plant species are coded as follows: Os, *Oryza sativa*; Ob, *O. brachyantha*; Sb, *Sorghum bicolor*; Zm, *Zea mays*; Si, *Setaria italic*; Ph, *Panicum hallii*; Ta, *Triticum aestivum*; Tu, *T. urartu*. The studying *TaDr1* accession is indicated in Bold.

The phylogenetic tree was constructed based on a BLASTX search for molecular similarity for the *TaDr1* protein (BC000036325) in cereal plant species and a group of TFs TaNF-YB for the comparison from NCBI Database. The sequences of all Dr1 proteins are distinct from all TaNF-YB TFs. Among Dr1 sequences, bread wheat (*Triticum aestivum*) and the diploid progenitor of A genome (*T. urartu*) form the first sub-clade; and cultivated rice (*Oryza sativa*) and closely related native grass from tropical Africa (*O. brachyantha*) are isolated in the second sub-clade. All other cereal species are joined together in the third sub-clade including sorghum (*Sorghum bicolor*), maize (*Zea mays*), foxtail millet (*Setaria italica*), and Hall’s panicgrass (*Panicum hallii*) (Figure 3).

### Expression Analysis of the *TaDr1* in Leaves of Control Plants and Plants Exposed to Drought

**Figure 4:**
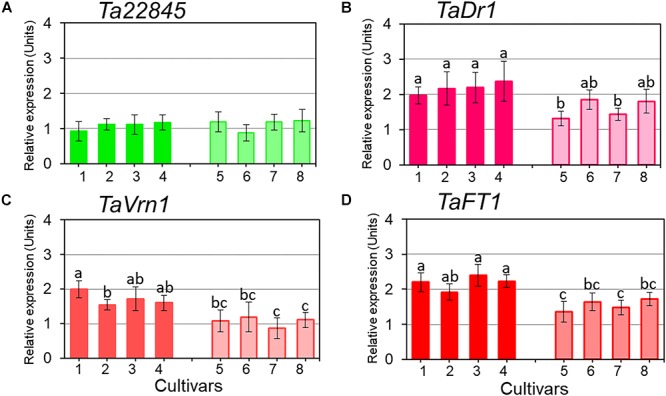
Expression of the reference gene *Ta22845* (ATP-dependent 26S proteasome, regulatory subunit) and target genes, *TaDr1*, *TaVrn1*, and *TaFT1*, in leaves of eight wheat cultivars in response to drought. The expression levels of *Ta22845* **(A)**, *TaDr1* **(B)**, *TaVrn1* **(C)**, and *TaFT1* **(D)** were calculated under drought relative to the corresponding controls in well-watered conditions. Eight wheat cultivars were studied, high-yielding are shown as darker boxes (**1.** Aktyubinka; **2.** Albidum 188; **3.** Altayskaya 110; and **4.** Saratovskaya 60), and the four low-yielding cultivars are shown as framed light filled boxes (**5.** Vera; **6.** Volgouralskaya; **7.** Yugo-Vostochnaya 2; and **8.** Zhenis). With the exception of Panel **A**, expression data were normalized using the averages of two reference genes, *Ta22845* and *Ta54825* (Actin), and presented as the average ± SE of three biological and two technical replicates for each genotype, experiment and treatment. Different letters above the bars indicate significant differences (*p* < 0.05) within each experiment calculated using ANOVA.

Expression profiles for *TaDr1* were recorded as the total of all three homeologous genes, *TaDr1A*, *TaDr1B* and BC000036325 using primers designed for the most conserved regions of these genes. Reference genes used in this study were stable across all genotypes in control and treatment conditions (Figure 4A). In plants exposed to drought, our results revealed significant up-regulation of *TaDr1* in all eight studied wheat cultivars (Figure 4B). Four high-yielding cultivars increased production of *TaDr1* transcripts 2–2.4 fold, while expression levels in plants of low-yielding cultivars were also increased compared to controls but not as strongly as in plants of high-yielding cultivars (Figure 4B).

Both flowering time regulators, *TaVrn1* and *TaFT1*, showed drought responsive expression similar to the expression of *TaDr1*. High-yielding cultivars (1–4) had higher expression levels of *TaVrn1* and *TaFT1* than low-yielding cultivars (5–8), although differences for some cultivars were not significant. These results show genotype-dependent co-expression following the same trend in all three studied genes, *TaDr1*, *TaVrn1*, and *TaFT1*, in leaves of plants grown under drought (Figure 4B–D).

Statistical analysis using Tests of Between-Subjects Effects for the gene expressions presented in Figure 4B–D shows a very low correlation between groups of high-yielding cultivars (1–4) and low-yielding cultivars (5–8), with *R*^2^ = 0.081, 0.123 and 0.118, respectively. In contrast, strong correlations (*R*^2^ = 0.897 and *R*^2^ = 0.957) were found between cultivars within each group, 1–4 and 5–8, for the three studied genes *TaDr1*, *TaVrn1*, and *TaFT1*, respectively (Table 2).

**Table 2:** Correlation analysis between groups of high-yielding and low-yielding cultivars for expression of the three genes, *TaDr1*, *TaVrn1*, and *TaFT1* (right column), and between cultivars within each group (bottom row).

	High-yielding cultivars	Low-yielding cultivars	*R*^2^
*TaDr1*	2.17 ± 0.08	1.60 ± 0.15	0.081
*TaVrn1*	1.72 ± 0.10	1.06 ± 0.08	0.123
*TaFT1*	2.18 ± 0.10	1.55 ± 0.10	0.118
****R******^2^**	0.897	0.957	

*Data represent the average of the relative expression units for four cultivars, with three biological replicates in each (*n* = 12) ± SE, extracted from Figure 4. The *R*^2^ correlation coefficient was calculated using Tests of Between-Subjects*.

## Discussion

Flowering time is a very important trait in wheat, and it was documented that earlier flowering by just a few days can increase the likelihood that plants can minimize the impact of terminal drought and ultimately improve their yield performance compared to wheat genotypes with later flowering times (Reviewed in: Shavrukov et al., 2017). Terminal or late season drought is the most common form of drought stress under most wheat production environments. In the current work, we compared the flowering time of four high-yielding and four low-yielding wheat cultivars and the expression of some genes related to flowering time. In a population of Recombinant breeding lines of durum wheat (*Triticum durum* Desf.) in diverse environments with drought, one QTL for heading date was identified in Chromosome 2A. However, this QTL had minimal or no effect on grain yield (Maccaferri et al., 2008). Different results were reported concerning early heading in synthetic bread wheat lines that correlated with higher grain yield under dry conditions compared to controls (Inagaki et al., 2007). The authors concluded that genes from the D genome could make an important contribution to the correlation in bread wheat, which is absent in tetraploid durum wheat.

The *TaDr1* gene was selected from a SNP database for genetic polymorphism analysis using molecular markers. This gene encodes a protein belonging to the group of general transcription repressors and is an important part of the plant regulatory system.

Two of the three homologous genes, *TaDr1A* and *TaDr1B*, were identified earlier in wheat (Stephenson et al., 2007), and a third *TaDr1* gene with the temporary name of contig BC000036325 identified in the current study, were localized in A, D and B genomes of bread wheat. Alignment of TaDr1 proteins with TaNF-YB3 reveals a high level of identity in the histone fold domain responsible for protein-protein and protein-DNA interactions (Figure 2). This result is in agreement with the previously published statement about the “high degree of similarity between TaDr1A, TaDr1B and TaNF-YB subunit members” (Stephenson et al., 2007).

The expression analysis of all three homeologous genes of *TaDr1* comprised an important part of the study of gene function, as published by Stephenson et al. (2007). However, analysis of the primer design for qPCR analysis of the genes, *TaDr1A* and *TaDr1B*, in Stephenson et al. (2007) did not reveal sufficient discrimination between these genes (Supplementary Material 2). One pair of primers published by Stephenson et al. (2007) was based on BT009234 and targeted the *TaDr1B* sequence for qPCR analysis, but it shows full consensus between the two genes, with no mismatches (indicated in green, Supplementary Material 2). Therefore, the use of these primers gave total (combined) expression for both genes, *TaDr1A* and *TaDr1B*. The second pair of primers, used and reported by Stephenson et al. (2007), was based on AF464903, where the reverse primer was again designed in the conserved region which is identical in both genes. Only a single nucleotide insertion and one SNP were found in the sequence of the TaDr1A-Fd primer (indicated in pink, Supplementary Material 2). We estimate that it contributes about 90–95% of the studied *TaDr1A* isoform specificity, so in the results presented by Stephenson et al. (2007), *TaDr1B* was over-estimated and represented the total expression of both genes combined, *TaDr1A* and *TaDr1B* (*TaDr1*).

In this context, we similarly measured total expression of all three homeologous genes *TaDr1* with qPCR primers based on the sequence BC000036325. Two mismatches at the 5′-end of the reverse primer (indicated in blue, Supplementary Material 2) can affect the specificity of the amplified mRNA of both genes, *TaDr1A* and *TaDr1B*, but only at an equal rate due to perfect consensus between AF464903 and BT009234 sequences in the primer-binding region.

In this work, the associations of an individual GoI with complex traits, such as flowering time and performance under drought, were studied in bread wheat cultivars. The regulatory gene, *TaDr1*, is clearly involved in the plant’s response to drought and its expression pattern correlates with the expression patterns of two other regulatory genes, *TaVrn1* and *TaFT1*, which are well-known regulators of flowering time. The existence of small differences in flowering time between high- and low-yielding wheat cultivars under moderate drought was also demonstrated.

In addition, over-expression of regulatory transgenes, *TaNF-YB4*, *TaDREB3*, or *TaSHN1*, as was shown in our earlier papers, activated sets of downstream genes and this led to significantly improved drought tolerance and/or increased grain yield of transgenic wheat plants (Yadav et al., 2015; Shavrukov et al., 2016a; Bi et al., 2018). These results confirm the relevance of the “single-gene for single-trait” approach in studying complex regulatory gene networks, such as, for instance, the response of bread wheat under limited water conditions.

The eight local wheat cultivars from Kazakhstan used in our study were separated into two groups representing high- and low-yielding varieties in the dry conditions of Northern and Central Kazakhstan, as discussed in our previous paper (Shavrukov et al., 2016b) and confirmed in the current study (Table 1). Under drought, the two groups of wheat cultivars showed quite variable expression profiles of *TaDr1*, with 2–2.4-fold and 1.3–1.8-fold higher expression of *TaDr1* in the first and second groups of cultivars, respectively (Figure 4B). The expression of *TaDr1*, identified as *TaDr1B* in cv. Babax (Stephenson et al., 2007), was reported to be about 2.3-fold above the level of controls, which is close to the highest level of the first group of wheat cultivars in the current study.

Our results indicate that the expression of *TaDr1* is dependent on wheat genotype. Four high-yielding cultivars showed very high expression of *TaDr1*, while gene expression was moderate in all four low-yielding cultivars compared to controls under drought treatment.

The two TFs, *TaVrn1* and *TaFT1*, are well studied and are known to strongly regulate the flowering time trait in wheat.Abiotic stresses, such as drought, can affect plant growth and development including flowering. In our recent paper, we reported that the *TaNFYC-A7* gene was differentially expressed under drought in the same cultivars studied here (Zotova et al., 2018). It is suggested that the TaDr1 protein could bind one or both of the TaNF-YB and TaNF-YC type subunits and consequently prevent their interactions or binding to the third subunit, TaNF-YA. It can therefore act as a repressor of the trimeric NF-Y transcription factor. We can extend this hypothesis and speculate that TaNF-Y, which is affected (deactivated) by TaDr1, can release the activity of *TaVrn1* and *TaFT1* promoters. This in turn leads to earlier flowering and ultimately improved performance of wheat genotypes grown in the dry environment of Northern and Central Kazakhstan. The proposed signaling pathway from *TaDr1* to *TaVrn1* and *TaFT1* is supported by the three genes’ co-expression results in the current study in wheat plants under drought. High expression of *TaDr1* was accompanied by significant up-regulation of *TaVrn1* and *TaFT1* transcripts. In experiments with drought stress, co-expression patterns in *TaDr1*, *TaVrn1*, and *TaFT1* were genotype-dependent and highly correlated, being much stronger in the four high-yielding wheat cultivars and less pronounced, but still significant, in the four low-yielding cultivars. Further strong evidence will be required to support or reject this hypothesis, including direct “protein-protein” interactions in the studied wheat genotypes.

The application of the Amplifluor-like SNP marker, KATU-W62, like other molecular markers, is a helpful tool for wheat genotyping of both modern cultivars and interspecific hybrids with wild relatives or species related to the genus *Triticum*. In this study, we were able to show that the markers can be deployed in tracking the different alleles in an F_3_ population resulting from a complex cross. This population will be used to assess the value of the marker in screening for enhanced drought tolerance under production conditions in Northern Kazakhstan. If our hypothesis is correct, we expect lines carrying the “*a*” allele to perform better under drought, with the strongest improvement shown for homozygotes “*aa*” in the presented study.

Identification of the *TaDr1* alleles can result in a better understanding of genetic polymorphism in the control of down-stream genes, like *TaVrn1* and *TaFT1*, which regulate vernalisation and flowering time. Together with the *Q* gene, the combined regulatory system can change the reproductive architecture of wheat plants and improve their tolerance to abiotic stresses, primarily drought.

## Author Contributions

LZ conducted the experiments with eight wheat cultivars and the genotyping with Amplifluor-like SNP analysis. AkK and SJ supervised experiments and interpreted results. NG supervised works with vernalisation and flowering time genes, and analysis of interspecific hybrid. NS, AzK, and AN conducted experiments with plant stresses and sampling. AO carried out work and analysis of interspecific hybrid. SS worked with plants in the field trial. VS coordinated experiments in the field. SL analyzed gene sequences in databases and wrote the corresponding section. CS analyzed results, and revised and edited the manuscript. CJ analyzed qRT-PCR data and revised the corresponding section. KS coordinated the qRT-PCR study and revised other sections. PL supervised the study and revised the final version of the manuscript. YS coordinated all experiments and wrote the first version of the manuscript.

## Conflict of Interest Statement

The authors declare that the research was conducted in the absence of any commercial or financial relationships that could be construed as a potential conflict of interest.

## Footnotes

1 http://www.cerealsdb.uk.net/cerealgenomics/CerealsDB

2 https://blast.ncbi.nlm.nih.gov

3 https://www.genome.jp/tools/blast

4 https://www.qiagenbioinformatics.com/products/clc-main-workbench

5 http://www.gramene.org

6 http://www.splitstree.org

7 http://biolabmix.ru/en/products

8 https://www.ncbi.nlm.nih.gov
