# Supplementary material for: The General Transcription Repressor TaDr1 Is Co-expressed With TaVrn1 and TaFT1 in Bread Wheat Under Drought
Source: Front Genet. 2019 Feb 8;10:63. doi: 10.3389/fgene.2019.00063 (PMC6375888; doi:10.3389/fgene.2019.00063)
Supplement: Supplementary file 1 [file Data_Sheet_1.PDF]

Supplementary material 1

Sequence of contig BC000036325 in bread wheat and annotated SNP from the cereals database (http://www.cerealsdb.uk.net/cerealgenomics/CerealsDB). The start-codon and Stop-codon of the identified gene are indicated in green and in red, respectively. The SNP position in the sequence was coded 'W', designating either nucleotides 'A' or 'T', and is highlighted in yellow. Two forward allele-specific primers and one common reverse primer are shown in Bold and highlighted in blue. Amplicon size is indicated. Sequences of primers and Universal probes are shown beneath. Specific 'tags' or 'tails' are in Italics. More details about allele-specific primers and Universal probes are available in our previous paper (Jatayev et al., 2017).

KATU-W62 - BC000036325

Contig sequence

ACGCCTAACCCTAACCCTAGCTACGATCCCCTTCTGCAACCCGGTCGTGTCTCAGGGCGCAT
CGCCGAAGGCCAAGCCCTCCCTCCCTAGCAGCGCCCGGACCCATCGATCCCGGCGGGACGCG
CCGTCGGCGCCCCAGGCTCAAGCTCCATCGCGCGCTCGACGGCGAGGGATGGATCCGATGGA
CATCGTGGGCAAGTCCAAGGAGGACGTCTCCCTCCCCAAATCAACAATGACCAAGATTATTA
AGGAGATGCTACCGCCTGATGTTCGAGTAGCAAGAGATACACAAGATCTTCTTGTTGAATGC
TGTGTAGAGTTCATCAATCTTCTTTCTTCGGAATCCAATGACGTGTGCAGCCGGGACGACAA
GAAGACTATTGCTCCTGAACATGTTATTAGGGCTCTGCAGGATCTTGGCTTCAAGGAGTACG
TTGAAGAAGTTTATGCAGCCTACGAACAGCACAAGCTTGAAACTCTGGACTCTCCAAAAGCA
ACCAAGTTCACTGGTATAGAGATGACTGAAGAAGAAGCTGTTGCGGAACAGCAGAGAATGTT
TGCTGAAGCCCGAGCAAGGATGAACAATGAAGCTGCCAAACCAAAGGAGCCTGCATTAGAAC
CACAGAATCAACCCCAACAGCCCCCACAACCTCAACTGCAGCTGCATCCTCAAGCACAGCAG
CCTCCACAACCCCAATCGCAACTGCATCATCCTCAATCACAGCAGCCCCTGCATCCTCAACT
GCAACCGTATACTCAGGCTCCACCACAGCAACCCCTGCATCCTCAACTGCAACCGTATACTC
AGGCTCCACCACAGCAACCCCTACAACCTCCACTGCAGCTGTATCCTCAGGCTCAACCTGAG
CAACCCCTGCAGCCTCAATCCTCAGGATCAACCACAGGAACCTGTGTAATCTCAACTGCAGC
TCCATCTACAACCGGCACCACTGCTGCTGCAACCGCCGCCCCAGCAATCCCCGCAATCTCAA
CTGCAGCTCCATCAGCAACCCCAGCCGACGCTAGTGCCGCCGCGGCCGCAACCTCAACCCCA
GCCACCTGAACTGCAGCAGCCCCAGCCGCTAACACAACTGCAAGCGGAACATGGCGTGGACT
GGACAGTTAGTGGTTCGGAACATGTAGCGTCACTATAAGTTAAGACTCTGCCTCCTTTAAAA
TTGTGCGTTAGGTTTGCCTGCATCTTGTACAATGTAAATTGA**GTGTGATTTCAGCCACCGTG**
TCTGTAATAATCTGAAGCTCTCTAGTAAGTGATGTACTTACTGTACTGGATATTGTGTTTAT
GACTGCTGT**WGTCTCATGGTATTGTGCGTGT**CGTGTCAGAAGCTACTCCATTACCAGTGTAA
TCAATTGCCTAACTTA

W = A/T

KATU-W62-SNP-F1: ACACGCACAATACCATGAGACT Tm=53C 45%GC
Rev.Comp. AGTCTCATGGTATTGTGCGTGT

KATU-W62-SNP-F2: ACACGCACAATACCATGAGACA Tm=53C 45%GC
Rev.Comp. TGTCTCATGGTATTGTGCGTGT

KATU-W62-SNP-R: GTGTGATTTCAGCCACCGTG Tm=53.8C 55%GC

PCR product size = 113 bp.

Ordered and used allele-specific primers:

KATU-W62-SNP-F1: *GAAGGTGACCAAGTTCATGCT***ACACGCACAATACCATGAGACT**
KATU-W62-SNP-F2: *GAAGGTCGGAGTCAACGGATT***ACACGCACAATACCATGAGACA**
KATU-W62-SNP-R: **GTGTGATTTCAGCCACCGTG**

Universal probe 1:
5′-FAM-AGCGATGCGTTCGAGCATCGC(T*-BHQ1)*GAAGGTGACCAAGTTCATGCT*-3′

Universal probe 2:
5′-VIC-AGGACGCTGAGATGCGTCC(T*-BHQ1)*GAAGGTCGGAGTCAACGGATT*-3′

Supplementary material 2

A fragment of the BLASTN comparison of three *TaDr1* gene sequences. Accession AF464903 was annotated in the NCBI database as the *TaDr1A* gene in bread wheat and accession BT009234 from NCBI database was identified as the *TaDr1B* gene by Stephenson et al. (2007). Accession BC000036325 was identified in the current study from Cereals DB (Supplementary material 1). Primers for qPCR analysis highlighted in pink and in green were designed and used for *TaDr1A* and *TaDr1A*, respectively, by Stephenson et al. (2007). Primers highlighted in blue for qPCR analysis for total expression of both genes, *TaDr1A* and *TaDr1B* (= *TaDr1*) were developed in the current study based on the sequence of BC000036325. The stop-codon of *TaDr1* is shown in red and the annotated SNP is shown in yellow. Mismatches are indicated in Bold and those in the designed primers are underlined.

```
AF464903       CCCAGCCGCTAACACAACTGCAAGCGGAACATGGCCTGGACTGGGACAGTTAGTGGTTCG
BT009234       CCCAGCCGCTAACACAACTGCAAGCGGAACATGGCCTGAACTGG-ACAGTTAGTGGTTCG
BC000036325    CCCAGCCGCTAACACAACTGCAAGCGGAACATGGCGTGGACTGG-ACAGTTAGTGGTTCG
               *********************************** ** ***** ***************

AF464903       GAACATGTAGCGTCACTATAAGTTAAGACTCTGCCTCCTTTAAAATTGTGCGTTAGGTTT
BT009234       GAACATGTAGCGTCACTATAAGTTAAGACTCTGCCTCCTTTAAAATTGTGCGTTAGGTTT
BC000036325    GAACATGTAGCGTCACTATAAGTTAAGACTCTGCCTCCTTTAAAATTGTGCGTTAGGTTT
               ************************************************************

AF464903       GCCTGCATCTTGTACAATGTAAATCGTGTGTGATTTCAGCCACCGTGTC--TAATAATCT
BT009234       GCCTGCATCTTGTACAATGTAAATCGTGTGTGATTTCAGCCACCGTGTCTCTAATAATCT
BC000036325    GCCTGCATCTTGTACAATGTAAATTGAGTGTGATTTCAGCCACCGTGTCTGTAATAATCT
               ************************ * **********************  *********

AF464903       GAAGCTCTCTAGTAAGCGATGTACTTACTGCGCTGGGTACTGTGTTTATGACTGCTGTAG
BT009234       GAAGCTCTCTAGTAAGCGATGTACTTACTGCGCTGGATACTGTGTTTATGACTGTTGTAG
BC000036325    GAAGCTCTCTAGTAAGTGATGTACTTACTGTACTGGATATTGTGTTTATGACTGCTGTWG
               **************** *************  **** ** ************** *** *

AF464903       TCTCATGGTATTGTGTGTGACGTGTCAGAAGCTACTCCATTACCAGTGTAATCAATTGCC
BT009234       TCTCATGGTATTGTGTGTGACGTGTCAGAAGCTACTCCATTACCAGTGTAATCAATTGCC
BC000036325    TCTCATGGTATTGTGCGTGTCGTGTCAGAAGCTACTCCATTACCAGTGTAATCAATTGCC
               *************** *** ****************************************

AF464903       TAACTTAATGTTCACCCGTGATGAT-----------------------
BT009234       TGACTTAATGTTCACCCGTGATGATAGTAATTGATTTCAGTGTGCTAA
BC000036325    TAACTTA-----------------------------------------
               * *****
```

Sequence of Reference genes used for qPCR

ATP-dependent 26S proteasome

Ta22845-F: GCTGGCTCGTTCAACTGATG

Ta22845-R: GGACCAAGCGTTCTGATTACTC

Size of amplicon: 202 bp (Paolacci et al., 2009)

Actin

Ta54825-F: TGACCGTATGAGCAAGGAG

Ta54825-R: CCAGACAACTCGCAACTTAG

Size of amplicon: 215 bp (Paolacci et al., 2009)
